# Low birth weight activates the renin–angiotensin system, but limits cardiac angiogenesis in early postnatal life

**DOI:** 10.14814/phy2.12270

**Published:** 2015-02-03

**Authors:** Kimberley C W Wang, Doug A Brooks, Brooke Summers-Pearce, Larisa Bobrovskaya, Darran N Tosh, Jaime A Duffield, Kimberley J Botting, Song Zhang, I Caroline McMillen, Janna L Morrison

**Affiliations:** 1 Early Origins of Adult Health Research Group, School of Pharmacy and Medical Sciences, Sansom Institute for Health Research, University of South AustraliaAdelaide, SA, Australia; 2 Mechanisms in Cell Biology and Disease Research Group, School of Pharmacy and Medical Sciences, Sansom Institute for Health Research, University of South AustraliaAdelaide, SA, Australia

**Keywords:** Angiogenesis, histone acetylation, low birth weight, renin–angiotensin system

## Abstract

Low birth weight (LBW) is associated with increased risk of adult cardiovascular disease and this association may be partly a consequence of early programming of the renin–angiotensin system (RAS). We investigated the effects of LBW on expression of molecules in the RAS and cardiac tissue remodeling. Left ventricular samples were collected from the hearts of 21 days old lambs that were born average birth weight (ABW) and LBW. Cardiac mRNA expression was quantified using real-time RT-PCR and protein expression was quantified using Western blotting. DNA methylation and histone acetylation were assessed by combined bisulfite restriction analysis and chromatin immunoprecipitation, respectively. There were increased plasma renin activity, angiotensin I (ANGI), and ANGII concentrations in LBW compared to ABW lambs at day 20. In LBW lambs, there was increased expression of cardiac ACE2 mRNA, decreased ANGII receptor type 1 (AT_1_R) protein, and acetylation of histone H3K9 of the *AT*_*1*_*R* promoter but no changes in AT_1_R mRNA expression and *AT*_*1*_*R* promoter DNA methylation. There was no difference in the abundance of proteins involved in autophagy or fibrosis. BIRC5 and VEGF mRNA expression was increased; however, the total length of the capillaries was decreased in the hearts of LBW lambs. Activation of the circulating and local cardiac RAS in neonatal LBW lambs may be expected to increase cardiac fibrosis, autophagy, and capillary length. However, we observed only a decrease in total capillary length, suggesting a dysregulation of the RAS in the heart of LBW lambs and this may have significant implications for heart health in later life.

## Introduction

Epidemiological studies have shown that individuals with low birth weight (LBW) have an increased risk of hypertension and cardiac disease in adulthood (Barker et al. 1989; Leon et al. 2000). Low birth weight can be caused by placental insufficiency, maternal undernutrition, smoking, or maternal hypoxia (McMillen and Robinson 2005). Neonates and children who were LBW also had elevated plasma renin activity and angiotensin II (ANGII) concentrations (Miyawaki et al. 2006; Franco et al. 2008) and this may be involved in the association between LBW, hypertension, and cardiac disease in adult life. The maturation pattern of sheep cardiomyocytes is similar to the human, where heart growth in early gestation is due to cardiomyocytes proliferation. In late gestation, heart growth is due to hypertrophy of cardiomyocytes that are terminally differentiated before birth (Smolich et al. 1989; Burrell et al. 2003; Jonker et al. 2007; Botting et al. 2012). The impact of LBW on cardiac renin–angiotensin system (RAS) is less clear. There is little context in terms of RAS activity in the neonates as our study will be the first to investigate the effect of low birth weight on cardiac RAS in neonates.

In humans and sheep, plasma or local cardiac ANGII acts via the cardiac ANGII type 1 (AT_1_R) and type 2 (AT_2_R) receptors (Burrell et al. 2001; Zohdi et al. 2007) to induce cardiac hypertrophy (Paradis et al. 2000; Ichihara et al. 2001). In addition to cardiac hypertrophy, phosphorylation of protein kinase B (Akt) by ANGII can regulate cardiac capillary density (Shiojima et al. 2005). We have previously shown that there is left ventricular hypertrophy and increased IGF-2R mRNA expression (Wang et al. 2011), an increase in phosphorylated Akt, and alterations in glucose metabolism in the heart of LBW compared to average birth weight (ABW) lambs (Wang et al. 2013a). Such an increase in Akt has been associated with pathological cardiac hypertrophy because the volume of tissue supplied by each capillary increases (Batra and Rakusen 1992; Friehs et al. 2004), resulting in insufficient oxygen delivery to each cardiomyocyte and an adverse impact on heart function (De Boer et al. 2003). Capillary density is also regulated by the expression of proangiogenic genes such as fibroblast growth factor 2 (FGF2), baculoviral inhibitor of apoptosis repeat containing 5 (BIRC5), chemokine (C-C motif) ligand 2 (CCL2), vascular endothelial growth factor (VEGF), angiopoietin-1, and angiopoietin-2 (Tian et al. 2011).

ANGII also stimulates the production of myocardial extracellular matrix (ECM) through the activation of cardiac AT_1_R and AT_2_R, which results in ECM remodeling (Ichihara et al. 2001). ECM remodeling is mediated by matrix metalloproteinases (MMP) and tissue inhibitor of metalloproteinases (TIMP) (Visse and Nagase 2003) and involves the accumulation of cardiac collagen, leading to fibrosis (Abhayaratna et al. 2008).

In the heart, ANGII exerts a proautophagic effect through activation of AT_1_R, but this effect can be suppressed by the activation of AT_2_R (Porrello et al. 2009). Autophagy, is a homeostatic mechanism that is involved in intracellular recycling (De Meyer et al. 2010), and can be induced by several stimuli, including the RAS and AMP-activated protein kinase (AMPK). Under physiological conditions, autophagy maintains cardiac mass and function (De Meyer et al. 2010). In hypertrophic hearts, autophagic signaling can be decreased, which further increases cardiac mass (De Meyer et al. 2010). Autophagy signaling includes forkhead box O (FoxO) 1, FoxO3a (Hariharan et al. 2010), and beclin 1, which leads to the formation of autophagosomes containing light chain 3B (LC3B) and recruitment of lysosomes containing lysosomal-associated membrane protein 1 (LAMP 1) (Kang et al. 2011). This process involves sirtuin 1 (SIRT 1), which has been shown to inhibit AT_1_R (Miyazaki et al. 2008).

Studies have shown that maternal high-salt diet-induced LBW decreased methylation of the *AT*_*1*_*R* promoter and increased cardiac AT_1_R gene expression in rat fetuses (Ding et al. 2010), while maternal diet-induced LBW decreased methylation of the *AT*_*1*_*R* promoter in the adrenals (Bogdarina et al. 2007). Intrauterine growth restriction (IUGR) has been shown to affect histone modification in organs such as the liver (Tosh et al. 2010) and lungs (Joss-Moore et al. 2010), but so far, the status of histone modifications in the heart has not been reported. In this study, we hypothesized that LBW would result in activation of the cardiac RAS and that this activation will be associated with increased angiogenesis, remodeling, autophagy, and epigenetic modifications in the heart of lambs 21 days after birth.

## Materials and Methods

### Animals, surgery, and blood sampling

All procedures were approved by the University of Adelaide Animal Ethics Committee and complied with the Australian code of practice for the care and use of animals for scientific purposes. The authors have read, and the experiments comply with, the policies and regulations of *The Journal of Physiology* (Drummond 2009).

#### Average birth weight (ABW) lambs

A frequency distribution curve of birth weights from 45 Control Merino singleton lambs, born during a 5-year period (Duffield et al. 2009), was used to define the mean birth weight of 5.63 ± 0.67 kg. Twenty Merino ewes were used in the current study and lambs were classified as ABW if their birth weight was within 2 SD of this mean value (ABW: 4.9–6.7 kg, *n* = 13). No surgical procedures were performed on the ewes that carried the ABW lambs.

#### Low birth weight (LBW) lambs

To induce placental restriction and a birth weight below 2 SD from the mean of Controls (i.e., below 4.9 kg; low birth weight, LBW, *n* = 8), nonpregnant ewes underwent surgery to remove the majority of endometrial caruncles from the uterus, leaving 3–8 caruncles in each horn (Danielson et al. 2005; Duffield et al. 2009; Dyer et al. 2009). After recovery, the ewes were mated and allowed to deliver spontaneously. Ewes and lambs from both groups were housed, fed, and maintained under the same conditions from a month before mating until tissue collection.

Venous blood samples from lambs were collected after ∼60 min of nonsuckling, on alternate days, between 9 am and 1 pm, and centrifuged at 1500 *g* for 10 min, before storage at −20°C (Duffield et al. 2009).

### Postmortem and tissue collection

On postnatal day 21, lambs were humanely killed with an intravenous overdose of sodium pentobarbitone (Virbac Pty Ltd, Milperra, NSW, Australia). The body, heart, and the left ventricle were weighed. Samples of the left ventricle were collected from each animal and frozen in liquid nitrogen or fixed in 4% paraformaldehyde. Information on the phenotype of the lambs including sex-specific changes in gene expression in fat (Duffield et al. 2009) and studies of cardiac growth (Wang et al. 2011) and metabolism (Wang et al. 2013a) have previously been described. There were no changes in heart weight and right ventricle between groups but the LBW lambs had a greater left ventricular weight relative to body weight (ABW, 3.34 ± 0.12 g kg^−1^; LBW, 3.78 ± 0.12 g kg^−1^; *P *<* *0.05 (Wang et al. 2011)).

### Plasma renin, ANGI, and ANGII radioimmunoassays

Renin activity (ABW, *n* = 12; LBW, *n* = 7), ANGI (ABW, *n* = 12; LBW, *n* = 7), and ANGII concentration (ABW, *n* = 11; LBW, *n* = 6) in EDTA-anticoagulated plasma samples from lambs at 20 days of age were determined by radioimmunoassay (ProSearch International, Malvern, VIC, Australia) (Zohdi et al. 2007; Wang et al. 2012c). Briefly, renin activity was determined in plasma samples remaining after previous analysis (Duffield et al. 2009) using a radioimmunoassay (Johnston et al. 1971) to measure ANGI in plasma after incubating with angiotensinase and angiotensin-converting enzyme inhibitors at 37°C and pH 6.2 for 1 h. Plasma renin activity was reported in ng mL^−1^ h^−1^ of ANGI generated after subtracting the blank (endogenous ANGI) at 0°C. The intra-assay CV was 10% and the inter-assay CV was 13%.

Plasma ANGII concentration was measured by radioimmunoassay. Samples were equilibrated for 20 h at 4°C with antibody raised in rabbit against ANGII which was n-terminally conjugated to bovine thyroglobulin. Monoiodinated ^125^I-ANGII (10,000 cpm in 100 *μ*L) was added and allowed to equilibrate for a further 16 h at 4°C before bound and free phases were separated using Dextran 10-coated charcoal and centrifugation. The sensitivity of the assay was 3.5 pg mL^−1^ and the intra-assay and inter-assay CV was 6.4% and 12%, respectively.

### RNA extraction and quantitative real-time RT-PCR

RNA was isolated from the left ventricle (∼100 mg) of each lamb (ABW, *n* = 12; LBW, *n* = 7) and cDNA was synthesized as previously described (Wang et al. 2011). Controls containing no Superscript III (NAC) and no RNA transcript (NTC) were used to test for genomic DNA and reagent contamination, respectively.

The reference genes (hypoxanthine phosphoribosyltransferase 1 (Passmore et al. 2009), phosphoglycerate kinase 1 (Passmore et al. 2009) and peptidylprolyl isomerase A (Passmore et al. 2009)) were chosen from a suite of reference genes, including: ribosomal protein P0 (Duffield et al. 2009), beta actin (Passmore et al. 2009), glyceraldehyde-3-phosphate dehydrogenase (Passmore et al. 2009), beta-2-microglobulin (B2M, Table1), tyrosine 3-monooxygenase (YWAHZ, Table1) based on expression analysis using the geNorm component of the qBase relative quantification analysis software (Hellemans et al. 2007), because their expression was stable across samples (Soo et al. 2012). The relative expression of mRNA transcripts of angiotensinogen (Table1), ANG-converting enzyme (ACE; Table1), ACE2 (Table1), AT_1_R (Table1), AT_2_R (Table1), FGF2 (Tian et al. 2011), BIRC5 (Tian et al. 2011), CCL2 (Tian et al. 2011), VEGF (Kasimanickam et al. 2010), angiopoietin-1 (Hagen et al. 2005), angiopoietin-2 (Ma et al. 2010), transforming growth factor (TGF) *β*_1_ (Bland et al. 2007), collagen type II (Table1), collagen type III (Lo et al. 2003), MMP 2 (Gallagher et al. 2007), TIMP 1 (Lo et al. 2003), TIMP 2 (Huang et al. 2010), TIMP 3 (Huang et al. 2010), and the reference genes were measured by qRT-PCR using Fast SYBR® Green Master Mix (Applied Biosystems, Mulgrave, VIC, Australia) in a final volume of 6 *μ*L on a ViiA7 Fast Real-time PCR system (Applied Biosystems) as previously described (Soo et al. 2012).

**Table 1:** Sequences of oligonucleotide primers used for quantitative real-time RT-PCR

Accession no.	Gene	Forward (F) and reverse (R) primer sequences
NM_001009284	B2M	F- CCGCCAGAAGATGGAAAGCCAAAT
		R- ACTGATCCTTGCTGTTGGGAGTGA
AY970970	YWAHZ	F- TGTAGGAGCCCGTAGGTCATCT
		R- TTCTCTCTGTATTCTCGAGCCATCT
NM_001114082.1	Angiotensinogen	F- TCGCTGCTGAGAAGATCAACAGGT
		R- TTTCCTTGGAAGTGGACGTAGGCA
AJ920033.1	ACE	F- AATTGCCTTCCTGCCCTTTGGCTA
		R- CCAGCGTCAAAGTGGGTTTCGTTT
NM_001024502.2	ACE2	F- AGAACCAGTCCTGGGATGCAGAAA
		R- AGTCAGCATGGAGTTGTCCCAGAA
NM_001009744.1	AT_1_R	F- ATTCCAGAAGGTCTGCATCCAGGT
		R- AATTGTGCCTTCCAGCTTTGGGAC
S81979.1	AT_2_R	F- TGGCTTGTCTGTCCTCATTG
		R- GCTGACCACTGGGCATACTT
NM_001009400	Collagen II	F- AAGGCTGCAACCTGGATGCCATTA
		R- CCTTGCTCTTGCTGATGTACCAGT

Primers were validated to generate a single transcript as confirmed by the presence of one double stranded DNA product of the correct size and sequence. Controls containing no cDNA were included for each primer set on each plate to test for reagent contamination. Melt curve/dissociation curves were also run to check for nonspecific product formation. Amplification efficiency reactions were performed on five triplicate serial-dilutions of cDNA template for each primer set. Amplification efficiencies were determined from the slope of a plot of C_t_ (defined as the threshold cycle with the lowest significant increase in fluorescence) against the log of the cDNA template concentration (ranging from 1 to 100 ng). The amplification efficiency was close to 100%. Each sample was run in triplicate for target and reference genes. The reactions were quantified by setting the threshold within the exponential growth phase of the amplification curve and obtaining corresponding C_t_ values. DataAssist Software v3.0 (Applied Biosystems) (Hellemans et al. 2007) was used to find the 2^−ΔCT^, which shows the abundance of each transcript relative to the abundance of the three stable reference genes and is expressed as mean normalized expression (MNE).

### Protein extraction and Western blotting

Left ventricular tissue (ABW, *n* = 13; LBW, *n* = 8) was sonicated three times for 30 s at 50% amplitude at 4°C (John Morris Scientific, Wayville, SA, Australia) in sonication buffer (2% SDS, 2 mmol L^−1^ EDTA, 50 mmol L^−1^ Tris, pH = 6.8; 1 mL of buffer per 50 mg of tissue). Samples were then boiled for 5 min and centrifuged at 15,700 *g* for 20 min. Protein content of extracts was determined by bicinchoninic acid protein assay as previously described (Wang et al. 2011). Prior to Western blot analysis, 20 *μ*g protein subjected to SDS-PAGE and stained with Coomassie blue reagent (Thermo Fisher Scientific, Scoresby, VIC, Australia) to ensure equal loading of the proteins (Muhlhausler et al. 2009; Nicholas et al. 2013). The Coomasie blue reagent-stained gel showed equal loading of proteins across samples (data not shown). We, thus, knew that our samples were adjusted so that an equal amount of protein was loaded in each well. Then 5 and 10 *μ*g of the same protein sample were loaded onto each gel to confirm linearity of the chemiluminescent signal (Muhlhausler et al. 2009; Wang et al. 2012b, 2013a; Nicholas et al. 2013).

Proteins for all samples were loaded and transferred onto the same nitrocellulose membrane (Hybond ECL, GE Health Care, Silverwater, NSW, Australia), with the exception of phospho-Akt that was transferred onto a PolyScreen® Polyvinylidene Difluoride Hybridization transfer membrane (PerkinElmer, Glen Waverley, VIC, Australia). The membranes were then incubated with the respective primary antibody: AT_1_R (Santa Cruz Biotechnology, Inc., Dallas, CA), AT_2_R (Santa Cruz Biotechnology, Inc., TX), SIRT 1 (Abcam, Cambridge, UK), phospho-Akt (Ser473, Cell Signaling Technology, Inc., Danvers, MA), forkhead box subfamily O (FoxO) 1 (Cell Signaling Technology, Inc.), FoxO3a (Abcam), phospho-FoxO1/FoxO3a (Thr24/Thr32, Cell Signaling Technology, Inc.), phospho-AMPK*α* (Thr172, Cell Signaling Technology, Inc.), beclin 1 (Cell Signaling Technology, Inc.), LC3B (Cell Signaling Technology, Inc.), and LAMP 1 overnight with agitation at 4°C and subsequently detected by enhanced chemiluminescence and protein abundance was quantified by densitometry as described previously (Wang et al. 2012b; Nicholas et al. 2013). Briefly, to measure the densitometry of each sample, we subtracted the background of each individual lane for background correction. We did not normalize the expression level of each protein to a control protein (loading control) because we found that there is an effect of low birth weight on the expression of standard housekeepers in the heart, which has been confirmed by others (Wadley et al. 2013).

### Methylation analysis

DNA methylation within the *AT*_*1*_*R* promoter was analyzed from the left ventricle of each lamb (ABW, *n* = 13; LBW, *n* = 8) by combined bisulfite restriction assay (COBRA) (Xiong and Laird 1997; Zhang et al. 2010, 2013; Wang et al. 2011). DNA (∼2 *μ*g) from individual hearts was subjected to bisulfite conversion (Epitect, Qiagen, Chadstone Centre, VIC, Australia). PCR was performed on 100 ng of bisulfite-converted DNA using primers and conditions that amplified methylated and unmethylated templates with no bias. Forward primer was 5′-GATGGTTGTGGTATTATTTTTTTT-3′ and reverse primer was 5′-TCTAAAACAACTCCAAATTTATAAC-3′. The amplicon of a 168 bp fragment derived from the promoter (http://genome.ucsc.edu/, −175 ∼ −8 bp relative to the transcription start site) was examined and digested with BstBI or TailI (New England Biolabs, Ipswich, MA). The intensity of uncut and cut fragments was quantified using an Experion Automated Electrophoresis System (Bio-Rad Laboratories, Gladesville, NSW, Australia). Percentage of methylation at 2 CpG sites was estimated by measuring the ratio of cut to uncut PCR product. The percentage of methylation was validated using methylation standard controls (e.g. 0%, 50%, and 100%).

### Chromatin Immunoprecipitation (ChIP) assay

Genomic DNA associated with specific histone proteins was analyzed from the left ventricle of each lamb (ABW, *n* = 11; LBW, *n* = 7) by ChIP assay. Nuclei were extracted from frozen left ventricle tissue with a SIGMA Nuclei Pure Prep Isolation Kit (Sigma-Aldrich Pty. Ltd., Sydney, Australia). The tissues were firstly homogenized using a dounce in a lysis master mix (nuclei PURE lysis buffer, 0.1 mol L^−1^ DTT and 10% Triton X-100) then filtered through a 100 *μ*m and 70 *μ*m cell strainer and centrifuged at 30,000 *g* for 45 min at 4°C through a sucrose solution to obtain isolated nuclei. Nuclei were resuspended in a micrococcal nuclease (MNase) buffer and incubated with MNase enzyme for 6 min before stopping the enzyme with 0.5M EDTA to obtain sheared chromatin fragments. Shearing size for chromatin of 150–600 bp was confirmed by agarose gel electrophoresis. Histone-specific DNA was then isolated from the chromatin extracts using a Qiagen ChIP OneDay kit (Qiagen). Chromatin was precleared then incubated with the target or control antibody H4K8ac, H3K9ac, RNA Pol III (positive control), or Rabbit IgG (negative control, Merck Milipore, Bayswater, VIC, Australia) on a rotating wheel at 4°C for 2 h. Samples were then mixed with Protein A beads and were rotated again at 4°C for 1 h and then washed five times with IP wash buffer and stored at 4°C. DNA was then isolated and purified from the protein/DNA immunoprecipitated samples using DNA Spin Columns (Qiagen). The relative expression of *ACE* promoter (Table2), *AT*_*1*_*R* promoter (Table2), *B2M* promoter (Table2), *YWAHZ* promoter (Table2), and *ubiquitin C* (*UBC*) promoter (Table2), which acted as housekeeper genes, associated with specific antibody binding to genomic DNA was then analyzed by qPCR on a ViiA7 Fast Real-time PCR system (Applied Biosystems).

**Table 2:** Sequences of oligonucleotide primers used for ChIP assay

Gene	Forward (F) and reverse (R) primer sequences
ACE promoter	F- CACGGGCAGTCGGTTCC
	R- GTCAGTGTGGCCTCATCCAT
AT_1_R promoter	F- GTAAGTGACACCGGGGATGG
	R- TTTATAGCGAGGGGCGTTGC
B2M promoter	F- TGATGTACAGGCAGCGAAGG
	R- ATTCAGGCAGCCAATCGGAA
YWAHZ promoter	F- GAAGGGTTGCGGGACATC
	R- ACTTCTCTACTCCTGTCCTGAG
UBC promoter	F- TTGTGCCTCAGAGCAGACAC
	R- TTCTGCGGTGATTTTCCCGA

### Determination of total length of capillaries in the left ventricle

Two nonconsecutive sections (5 *μ*m) of paraffin-embedded samples of the left ventricle from each animal (ABW, *n* = 8; LBW, *n* = 6) were stained with anti-actin (*α*-smooth muscle antibody; Sigma-Aldrich, MO), to stain pericytes that were wrapped around the capillaries (Hirschi and D'Amore 1996; Bergers and Song 2005; Botting et al. 2014). Forty counting frames (area 40,000 *μ*m^2^; ×600 magnification) were randomly selected per section and visualized using NewCAST software (Visiopharm, Hoersholm, Denmark) and a digital camera DP72 (Olympus Australia Pty. Ltd, Notting Hill, VIC, Australia) connected to a BX53 Research Microscope (Olympus Australia Pty. Ltd). Point counting was used to determine the total number of capillary profiles (∑*Q*^*−*^) falling within counting frames and the area of the reference space (sum of points hitting ventricular tissue (∑*P*) multiplied by the area of each point (area of counting frame/4)). Counters were blinded, with respect to the sample being assessed. The total length of capillaries in the left ventricle (*L*(cap/left ventricle)) was calculated using the formula: *L*(cap/left ventricle) = 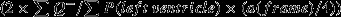
× volume of left ventricle (Saccà et al. 1994; Brüel et al. 2005)

### Left ventricular fibrosis

Paraffin-embedded samples of the left ventricle (ABW, *n* = 13; LBW, *n* = 8) were sectioned at 7 *μ*m and stained with Masson's Trichrome stain. Whole sections were examined for each animal to visualize fibrosis using AnalySIS software (Olympus Soft Imaging Solutions, Gulfview Heights, SA, Australia).

### Statistical analysis

The effects of treatment and sex on the absolute and relative weights of organs and the relative expression of genes/proteins were determined using a 2 way ANOVA (SPSS 18 for Windows, Statistical Package for Social Scientists Inc., St Leonards, NSW, Australia). There was no significant effect of sex and no interaction between treatment and sex for any of the measured parameters, which may be due to the study not having enough power to measure sex effect. Therefore, the effect of treatment (ABW vs. LBW) on plasma renin activity and ANGII concentration and expression of cardiac gene and proteins were analyzed using Student's t-tests. Birth weight is a continuous measure, therefore, linear regression using data pooled from both ABW (*n* = 13) and LBW (*n* = 8) lambs was used to determine the relationships between body weight, heart weight, left ventricle weight, or total length of capillaries and genes or proteins of interest. All data are presented as mean ± SEM. Analyses were performed using SPSS 18 for Windows (Statistical Package for Social Scientists Inc., IL) and a probability of <5% (*P *<* *0.05%) was considered significant.

## Results

### Increased circulating plasma renin activity, ANGI, and ANGII concentration but reduced cardiac AT_1_R protein expression in LBW lambs

**Figure 1:**
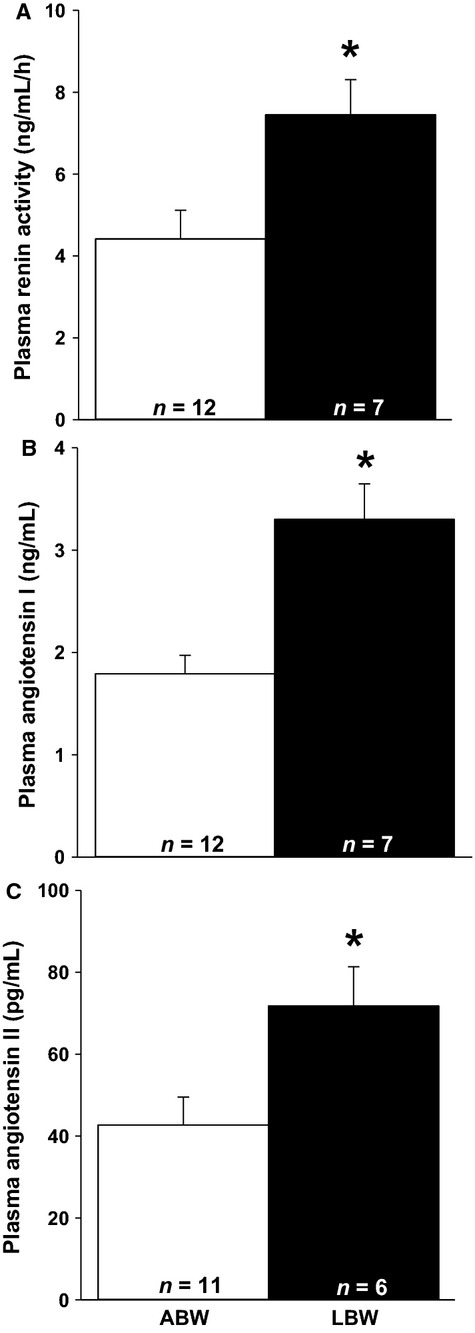
LBW increases plasma renin activity, ANGI, and ANGII concentration. There was increased plasma renin activity (A), plasma ANGI concentration (B), and plasma ANGII concentration (C) in the LBW compared to ABW lambs at postnatal day 20. Sample size for each group is indicated in the bar. *Significantly different from ABW lambs (*P *<* *0.05). ABW, average birth weight; LBW, low birth weight.

**Figure 2:**
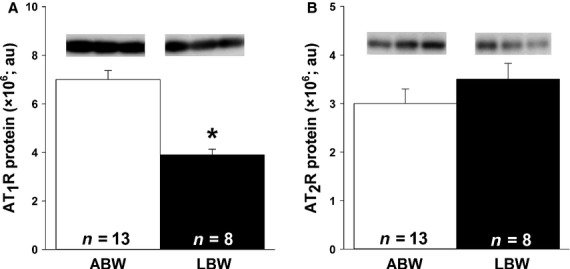
LBW resulted in decreased amount of cardiac AT_1_R but not AT_2_R protein. There was decreased AT_1_R (A), but not AT_2_R (B), protein in LBW compared to ABW lambs. All samples were loaded on the same blot and representative bands from three samples in each group are presented. Sample size for each group is indicated in the bar. *Significantly different from ABW lambs (*P *<* *0.05). ABW, average birth weight; LBW, low birth weight.

Plasma renin activity (Fig.1A), ANGI concentration (Fig.1B), and ANGII concentration (Fig.1C) were significantly higher in LBW compared to ABW lambs. There was a direct relationship between plasma renin activity and ANGII concentration (*P *<* *0.001, *r*^2^ = 0.560, ANGII concentration = 11.44 * (plasma renin activity-1.60)). ACE2 mRNA expression was higher in the hearts of LBW compared to ABW lambs (Table3). There were, however, no differences in cardiac angiotensinogen, ACE, AT_1_R, and AT_2_R mRNA expression between LBW and ABW lambs (Table3). Cardiac AT_1_R abundance was lower (Fig.2A), but SIRT 1 (ABW, 45880 ± 3417 au; LBW, 52889 ± 4415 au) and AT_2_R abundance (Fig.2B) were not different in LBW compared to ABW lambs.

**Table 3:** mRNA expression of components of the cardiac RAS in ABW and LBW lambs

mRNA (MNE)	ABW (*n* = 12)	LBW (*n* = 7)
Angiotensinogen	0.02 ± 0.003 (9)	0.02 ± 0.005 (6)
ACE	0.05 ± 0.01 (10)	0.04 ± 0.01
ACE2	0.002 ± 0.0003 (10)	0.005 ± 0.001 (6)*
AT_1_R	0.07 ± 0.004	0.06 ± 0.01
AT_2_R	0.07 ± 0.01	0.07 ± 0.01

Values are mean ± SEM (*n*).

* Significantly different from ABW lambs (*P *<* *0.05). ABW, average birth weight; LBW, low birth weight; MNE, mean normalized expression.

### No changes in methylation of cardiac *AT*_*1*_*R* promoter or acetylation of histones in the *ACE* promoter but decreased cardiac acetylation of histone H3K9 in the *AT*_*1*_*R* promoter

**Figure 3:**
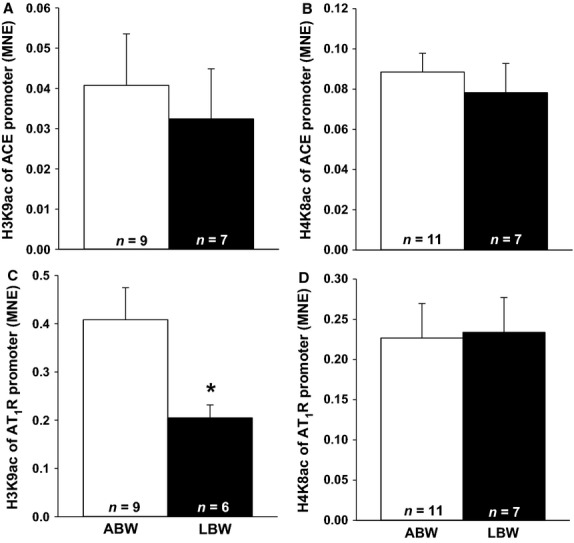
LBW decreased acetylation of histone H3K9 in the *AT*_*1*_*R* promoter. There were no changes in acetylation of histone H3K9 (A) and H4K8 (B) in the *ACE* promoter in LBW compared to ABW lambs. There was decreased acetylation of histone H3K9 (C) but no difference of histone H4K8 (D) in the *AT*_*1*_*R* promoter in LBW compared to ABW lambs. Sample size for each group is indicated in the bar. *Significantly different from ABW lambs (*P *<* *0.05). ABW, average birth weight; LBW, low birth weight.

There was no methylation present (0%) on the two CpG sites analyzed for the *AT*_*1*_*R* promoter in the heart of ABW and LBW lambs. There was also no change in acetylation of histone H3K9 (Fig.3A) or H4K8 (Fig.3B) in the *ACE* promoter. There was decreased acetylation of histone H3K9 (Fig.3C) but not H4K8 (Fig.3D) in the *AT*_*1*_*R* promoter.

### There was no relationship between abundance of ANGII receptors and left ventricular weight relative to body weight

There was a direct relationship between birth weight and AT_1_R protein in the heart (*P *<* *0.001, *r*^2^ = 0.546, AT_1_R protein = 1.20e^+6^(birth weight) − 3.26e^+5^)); but there was no relationship between left ventricular weight relative to body weight and AT_1_R protein in the heart (data not shown). There was no relationship between body weight, heart weight, absolute left ventricle weight, or left ventricle weight relative to body weight and the amount of AT_2_R protein in the heart (data not shown).

### Increased BIRC5 and VEGF mRNA expression but decreased total length of capillaries in the left ventricle of LBW lambs

**Figure 4:**
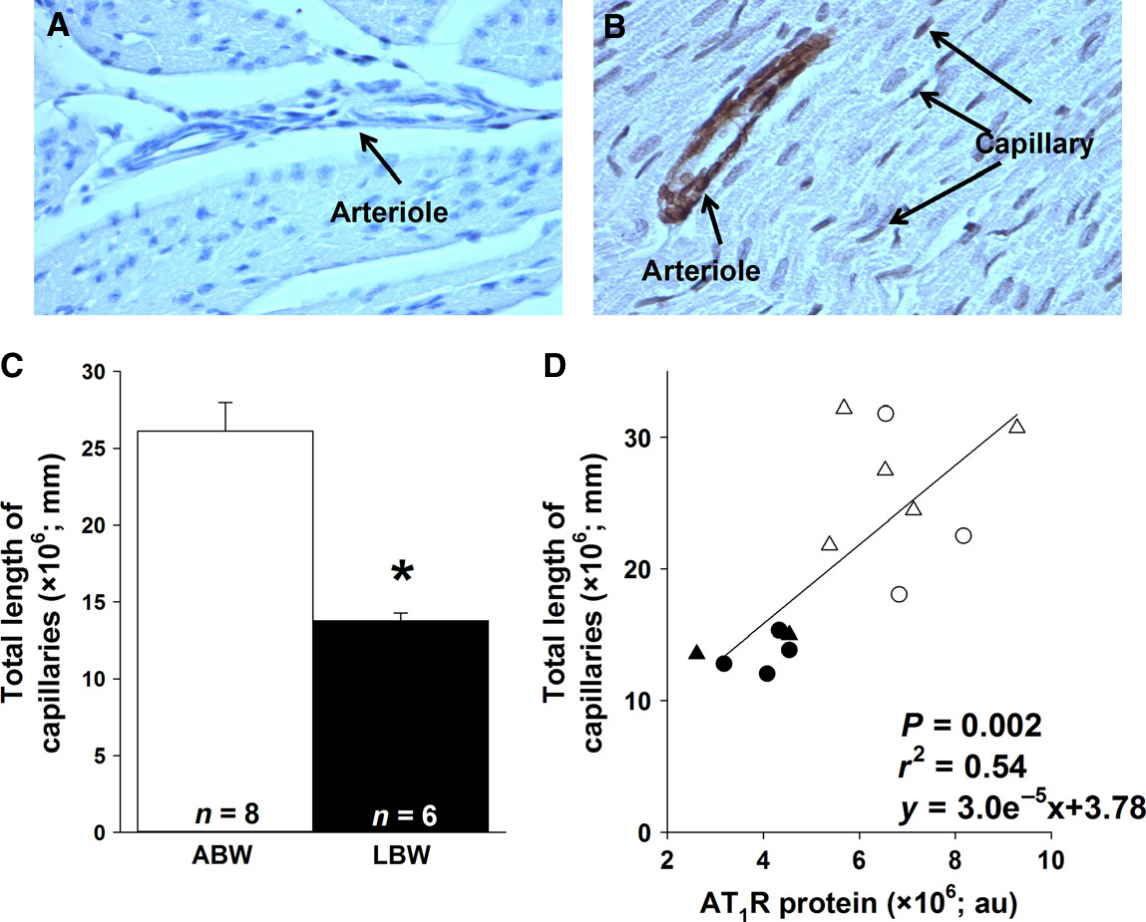
LBW decreased the total length of capillaries in the left ventricle. Representative light micrographs of a negative control (A) and capillary stained with anti-actin (*α*-smooth muscle antibody) left ventricle section (B) in LBW lambs (magnification: ×400). There was a lower total length of capillaries (mm) in LBW compared to ABW lambs (C). There was also a direct correlation between total length of capillaries (mm) and AT_1_R protein (D). Sample size for each group is indicated in the bar. Open triangles, male ABW (average birth weight) lambs; open circles, female ABW lambs; closed triangles, male LBW (low birth weight) lambs; closed circles, female LBW lambs. *Significantly different from ABW lambs (*P *<* *0.05).

There was an increase in the mRNA expression of cardiac BIRC5 and VEGF in the LBW compared to ABW lambs, but no difference in the mRNA expression of cardiac FGF2, CCL2, angiopoietin-1, or angiopoietin-2 between ABW and LBW lambs (Table4). Total length of capillaries in the left ventricle of the LBW lambs was lower compared to the ABW lambs (Fig.4A). There was a direct relationship between the amount of AT_1_R and total length of capillaries (Fig.4B), and an inverse relationship between total length of capillaries and phospho-Akt protein (*P *=* *0.01, *r*^2^ = 0.44, total length of capillaries = −0.01 (phospho-Akt protein) + 25.24).

**Table 4:** mRNA expression of factors associated with cardiac angiogenesis in ABW and LBW lambs

mRNA (MNE)	ABW (*n* = 12)	LBW (*n* = 7)
FGF2	0.08 ± 0.01	0.07 ± 0.01
BIRC5	0.03 ± 0.003	0.05 ± 0.01*
CCL2	0.01 ± 0.001	0.01 ± 0.001
VEGF	0.61 ± 0.11	1.61 ± 0.33*
Angiopoietin-1	0.04 ± 0.01	0.01 ± 0.003
Angiopoietin-2	0.19 ± 0.03	0.21 ± 0.05

Values are mean ± SEM.

* Significantly different from ABW lambs (*P *<* *0.05). ABW, average birth weight; LBW, low birth weight; MNE, mean normalized expression.

### Cardiac fibrosis was not present in the LBW lambs

There were no differences in the mRNA expression of cardiac TGF*β*_1_, collagen type II, collagen type III, MMP 2, TIMP 1, TIMP 2, and TIMP 3 in the LBW compared to ABW lambs (Table5). There was minimal interstitial collagen deposition and there was no measurable fibrosis in the hearts of either ABW or LBW lambs (data not shown).

**Table 5:** mRNA expression of factors associated with cardiac remodeling in ABW and LBW lambs

mRNA (MNE)	ABW (*n* = 12)	LBW (*n* = 7)
TGF-*β*_1_	0.15 ± 0.03	0.10 ± 0.01
Collagen type II	3.78 ± 0.55	5.48 ± 0.37
Collagen type III	0.34 ± 0.09	0.17 ± 0.02
MMP 2	0.40 ± 0.04	0.44 ± 0.02
TIMP 1	0.11 ± 0.01	0.09 ± 0.01
TIMP 2	1.18 ± 0.14	0.82 ± 0.08
TIMP 3	1.20 ± 0.14	0.84 ± 0.05

Values are mean ± SEM. ABW, average birth weight; LBW, low birth weight; MNE, mean normalized expression.

### Decreased cardiac FoxO1 and FoxO3a protein in LBW lambs, but no change in molecules involved in autophagy

**Figure 5:**
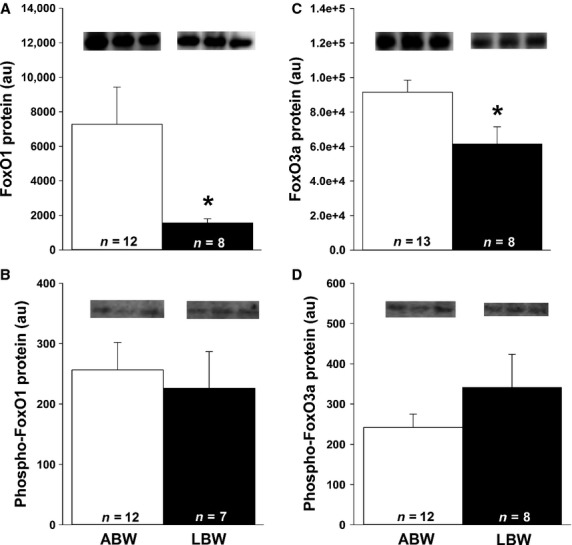
LBW decreased cardiac FoxO protein. There was decreased FoxO1 (A) and FoxO3a (C) protein, but not their phosphorylated forms (B and D) in the LBW compared to ABW lambs. All samples were loaded on the same blot and representative bands from three samples in each group are presented. Sample size for each group is indicated in the bar. *Significantly different from ABW lambs (*P *<* *0.05). ABW, average birth weight; LBW, low birth weight.

**Figure 6:**
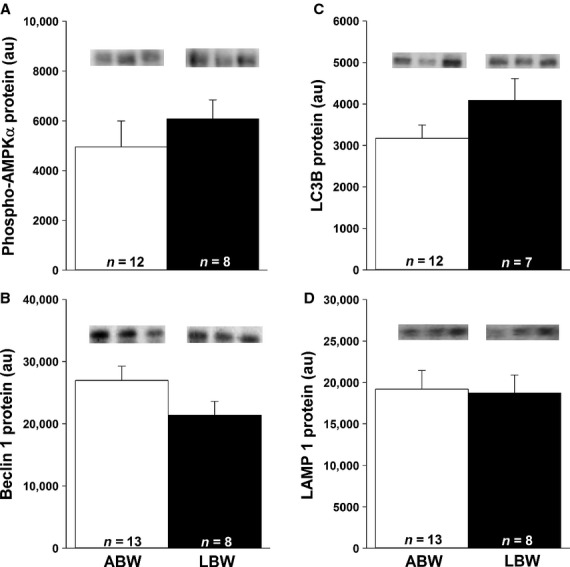
LBW had no effect on the cardiac autophagy. There were no changes in phospho-AMPK*α* (A), beclin 1 (B), LC3B (C), or LAMP 1 (D) abundance in LBW compared to ABW lambs. All samples were loaded on the same blot and representative bands from three samples in each group are presented. Sample size for each group is indicated in the bar. ABW, average birth weight; LBW, low birth weight.

Cardiac FoxO1 (Fig.5A) and FoxO3a (Fig.5C) were lower in LBW compared to ABW lambs. There was, however, no difference in phospho-FoxO1 (Fig.5B) or phospho-FoxO3a (Fig.5D) in the hearts of LBW compared to ABW lambs. There were also no differences in phospho-AMPK*α*, beclin 1, LC3B, and LAMP 1 (Fig.6A–D) protein in the hearts of the LBW compared to ABW lambs.

## Discussion

### LBW and plasma renin activity and ANGII concentration

In this study, LBW was associated with increased plasma renin activity and ANGII concentration. This is a programmed effect because it is consistent with the increase in RAS being responsive to chronic hypoxemia in fetal life and is maintained into postnatal life. In rat models of IUGR induced by glucocorticoid exposure or placental insufficiency, there was increased activity of hepatic and vascular RAS and it has been suggested that this may underlie the increased risk of hypertension in adult life (O'Regan et al. 2004; Grigore et al. 2007). However, in sheep models of IUGR induced either by 20 days of umbilicoplacental embolization (Zohdi et al. 2007) or twinning (De Matteo et al. 2008), there were no differences in plasma renin activity or ANGII concentrations when compared to controls in either fetal or postnatal life. It is possible that differences in the timing, duration, and degree of hypoxia and thus growth restriction between these models (Morrison 2008) may explain differences in the activation of the systemic RAS.

In this sheep model of IUGR there are documented changes in RAS in fetal life, including a greater hypotensive response to an ACE inhibitor (Edwards et al. 1999) and increased prostaglandin synthesis to maintain renin gene expression in the kidney (Williams et al. 2002). It is possible that the programming in the kidney is different than that in the heart and renal RAS is relevant in terms of the measured changes in the systemic concentrations of renin activity and ANGII. In fact, the renal renin mRNA expression was directly related to fetal arterial PO_2_ and is reduced in the placentally restricted fetus (Zhang et al. 2000). We have previously shown that some changes in gene expression that are observed in the placentally restricted fetus are maintained into postnatal life (IGF-2 and IGF-2R (Wang et al. 2011)), while others are not (IGF-1 and IGF-1R (Wang et al. 2011)). Therefore, future studies measuring gene expression of RAS in the kidney and nephron number in the LBW lambs is needed.

### Local RAS and cardiac hypertrophy in LBW lambs

The increased cardiac ACE2 mRNA expression in the LBW lamb may reduce the actions of increased plasma ANGII concentration in the heart because ACE2 counteracts the effects of accumulating ANGII by converting ANGII to the vasodilator ANG1-7 and by competing with ACE for ANGI (Oudit et al. 2003). Elevated ACE2 gene expression has been detected in rat models of myocardial infarction and in failing human hearts (Burrell et al. 2005), highlighting the role that ACE2 plays in opposing the effects of ANGII. Our plasma and AT_1_R protein data are consistent with a previous study showing that cardiac expression of both AT receptors is inversely related to circulating ANGII concentration (Sun and Weber 1993), which may reflect receptor downregulation (Yang et al. 1998). In that study, there was no change in DNA methylation, but we found decreased acetylation of histone H3K9 *AT*_*1*_*R* promoter and reduced AT_1_R protein but not mRNA expression in the hearts of LBW lambs. This is interesting because histone acetylation and gene expression are generally correlated (Struhl 1998), which is consistent with our finding for ACE where there was no change in histone acetylation or gene expression. Our finding of a decrease in AT_1_R protein abundance showed that the decrease in H3K9 acetylation did impact protein abundance. This is supported by a recent finding that protein expression is tightly correlated with histone acetylation (Wang et al. 2013b).

In the current study of LBW with left ventricular hypertrophy, there was no relationship between AT_1_R and the weight of the left ventricle. However, activation of either the AT_1_R or AT_2_R signaling pathways has been associated with cardiac hypertrophy (Ichihara et al. 2001; van Esch et al. 2010). It is possible that the increase in plasma renin, ANGI, and ANGII concentrations, together with increased ACE2 gene expression and decreased AT_1_R protein may be explained by a feedback mechanism to prevent ANGII-induced hypertrophy in cardiomyocytes. However, there may also be other signaling pathways that cause hypertrophy involved, such as IGF-2R (Wang et al. 2012a,b), while a consequence of reduced cardiac AT_1_R may be decreased activation of angiogenesis and thus a reduction in cardiac capillary density.

### LBW and total length of capillaries in the heart

Here, we have shown that phospho-Akt (Wang et al. 2013a), VEGF, and BIRC5 are elevated in the heart of the LBW lamb and that there is a decrease in total length of capillaries, suggesting that heart function in the LBW lamb is compromised as early as 21 days after birth. These findings are consistent with previous work showing that decreased capillary density is a feature of cardiac hypertrophy in the adult (Batra and Rakusen 1992). Interestingly, there were no differences in VEGF mRNA or total capillary length in the hearts of placentally restricted fetuses in late gestation compared to Control fetuses (Botting et al. 2014).These conflicting data between placentally restricted fetuses and LBW postnatal lambs may be due to the hearts of LBW lambs still in a process of adaptation or that LBW lambs may have relatively left ventricular hypertrophy with larger cardiomyocytes and low ratio of capillaries in the heart. We acknowledge that a limitation of this study is that we did not have access to perfusion-fixed heart tissue or a specific endothelial marker to stain for capillaries. Perfusion fixation opens the capillaries and combined with a specific marker of endothelial improves the ability to identify capillaries. Specific markers of endothelial cells have been successful on snap-frozen sections of sheep tissue but with limitations in the amount of available tissue for this study, we were unable to use snap-frozen heart samples for immunohistochemistry. We have, however, stained the fixed heart samples with antiactin (*α*-smooth muscle antibody) to stain the pericytes that wrap around capillaries and reported this in other study (Botting et al. 2014).

Shiojima et al. have suggested that factors from both the vasculature and cardiomyocytes cross talk to regulate cardiac angiogenesis, contractile function, and heart size (Shiojima et al. 2005). ANGII activation of AT_1_R is important for coronary capillary angiogenesis (Jesmin et al. 2002). However, transgenic mice with overexpression of cardiac ANGII have decreased capillary density (Xu et al. 2007). On the other hand, capillary density is reduced in aged rats with increased left ventricular mass (Batra and Rakusen 1992), and long-term cardiac Akt activation leads to pathological cardiac hypertrophy (Shiojima et al. 2005). In a model of pregnancy at high altitude, resulting in fetal chronic hypoxemia in late gestation, there was no difference in cardiac capillary density just before birth, however, these animals were not growth restricted (Lewis et al. 1999). In addition, rats at 10 weeks of age that were exposed to chronic hypoxia in utero had increased cardiac contractility performance, but decreased capillary density (Hauton and Ousley 2009). Thus, it may be that reduced oxygen in utero, depending on the degree, duration, and timing of the insult (Morrison 2008), affects cardiac angiogenesis and subsequently cardiac health.

### LBW and cardiac ECM remodeling

Cardiac fibrosis was not present in the LBW lambs at 21 days after birth. IUGR has been associated with increased aortic wall thickening, fibrosis (Briscoe et al. 2004), and increased collagen gene expression in the heart (Xu et al. 2006). Adult offspring of dams exposed to maternal protein restriction had increased cardiac fibrosis, but not capillarization (Lim et al. 2006), while chickens exposed to chronic hypoxia during embryonic development had increased cardiac collagen content (Tintu et al. 2009). These studies provide evidence that reduced substrate (oxygen and/or nutrients) supply in utero may affect postnatal cardiac ECM integrity. In addition, increased cardiac AT_1_R concentration has been associated with increased fibrosis (Xu et al. 2007). The findings of fibrosis in hearts of LBW animals of these studies may be due to hypertension; however, other studies have shown that LBW lambs have the same blood pressure as ABW lambs at 1 year of age (Owens et al. 2007). The fibrosis may also be the result of an adaptation to fewer cardiomyocytes (Corstius et al. 2005) because it has been shown that there is a relationship between birth weight and cardiomyocyte number in twins (Stacy et al. 2009).

### ANGII receptors and cardiac autophagy in LBW lambs

The similar amounts of cardiac phospho-AMPK*α* and SIRT 1 protein for LBW and ABW lambs may help explain the lack of change in the downstream autophagy signaling pathway. ANGII can regulate cardiac autophagy and AT_2_R constitutively antagonizes AT_1_R-mediated autophagy (Porrello et al. 2009). FoxO protein function and transcriptional activity are affected by different posttranslational modifications including deacetylation of FoxO1 and FoxO3a by SIRT 1, which is essential in mediating starvation-induced autophagy (Salminen and Kaarniranta 2009; Hariharan et al. 2010). SIRT 1 inhibits AT_1_R (Miyazaki et al. 2008) and is also required in the formation of autophagic vacuoles through acetylation of the autophagy-related (Atg) proteins (Salminen and Kaarniranta 2009). Under conditions of amino acid or glucose deprivation, AMPK is activated and has the ability to phosphorylate FoxO3a and upregulate Atg proteins (Chiacchiera and Simone 2009). Although our results suggest that autophagy may not be different between groups, additional methods such as transmission electron microscopy are needed to confirm this finding (Martinet et al. 2013).

## Conclusion

**Figure 7:**
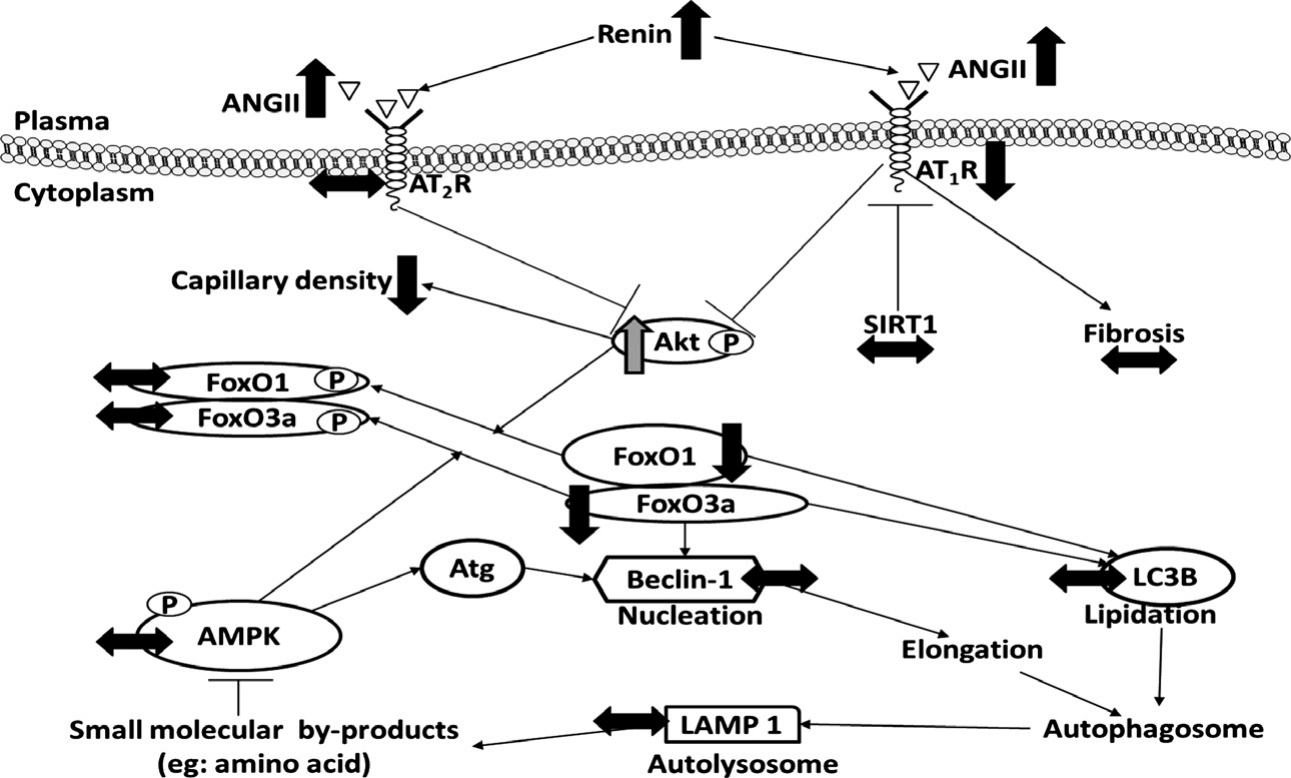
LBW affects cardiac RAS but not cardiac autophagy. Decreased activation of the AT_1_R did not change the amount of downstream proteins in the autophagy signaling pathway. (↑), increased protein expression in LBW compared to ABW lambs; (↓), decreased protein expression in LBW compared to ABW lambs; (↔), no changes in protein expression in LBW compared to ABW lambs; (p), protein phosphorylation.

Our study has shown that in LBW lambs, there was a decrease in cardiac AT_1_R in the presence of higher circulating renin activity, ANGI, and ANGII concentration, but that there were no changes in cardiac autophagy or fibrosis in the neonatal lamb (Fig.7). The total length of coronary capillaries was, however, lower in the LBW lambs (Fig.7), which may lead to impaired nutrient and oxygen supply to the hypertrophic heart. Our data therefore suggest that small size at birth has a pathological impact on cardiac signaling pathways, which may explain the risk of adult cardiovascular disease in later life. Future studies using pharmacological inhibitors to reduce activation of the RAS in the LBW lamb and its impact on capillary density are needed.
